# Report of the first international workshop on onchocerciasis-associated epilepsy

**DOI:** 10.1186/s40249-018-0400-0

**Published:** 2018-03-22

**Authors:** Robert Colebunders, Michel Mandro, Alfred K. Njamnshi, Michel Boussinesq, An Hotterbeekx, Joseph Kamgno, Sarah O’Neill, Adrian Hopkins, Patrick Suykerbuyk, Maria-Gloria Basáñez, Rory J. Post, Belén Pedrique, Pierre-Marie Preux, Wilma A. Stolk, Thomas B. Nutman, Richard Idro

**Affiliations:** 1 Global Health Institute, University of Antwerp, Antwerp, Belgium; 2 Provincial Health Division of Ituri, Bunia, Democratic Republic of the Congo; 3 Department of Neurology, Yaoundé Central Hospital/University of Yaoundé 1, Brain Research Africa Initiative (BRAIN), Yaoundé, Cameroon; 4 Institut de Recherche pour le Développement (IRD), Montpellier, France; 5 Centre for Research on Filariasis and other Tropical Diseases (CRFilMT), and Faculty of Medicine and Biomedical Sciences, University of Yaoundé 1, Yaoundé, Cameroon; 6 Department of Public Health, Institute of Tropical Medicine, Antwerp, Belgium; 7 Neglected and Disabling Diseases of Poverty Consultant, Kent, UK; 8 London Centre for Neglected Tropical Disease Research, Imperial College London, London, UK; 9 London School of Hygiene & Tropical Medicine and Liverpool John Moores University, London, UK; 10 Drugs for Neglected Diseases initiative, Geneva, Switzerland; 11 Preux Pierre-Marie, INSERM, University Limoges, CHU Limoges, UMR_S 1094, Tropical Neuroepidemiology, Institute of Neuroepidemiology and Tropical Neurology, CNRS FR 3503 GEIST, 87000 Limoges, France; 12 Department of Public Health, Erasmus MC, University Medical Centre Rotterdam, Rotterdam, The Netherlands; 13 Laboratory of Parasitic Diseases, National Institutes of Health, Bethesda, Maryland USA; 14 Makerere University, College of Health Sciences, Kampala, Uganda

**Keywords:** Onchocerciasis, Epilepsy, Nodding syndrome, Nakalanga syndrome, Prevalence, Burden of disease, Africa

## Abstract

**Background:**

Recently, several epidemiological studies performed in *Onchocerca volvulus*-endemic regions have suggested that onchocerciasis-associated epilepsy (OAE) may constitute an important but neglected public health problem in many countries where onchocerciasis is still endemic.

**Main text:**

On October 12–14^th^ 2017, the first international workshop on onchocerciasis-associated epilepsy (OAE) was held in Antwerp, Belgium. The workshop was attended by 79 participants from 20 different countries. Recent research findings strongly suggest that *O. volvulus* is an important contributor to epilepsy, particularly in meso- and hyperendemic areas for onchocerciasis. Infection with *O. volvulus* is associated with a spectrum of epileptic seizures, mainly generalised tonic-clonic seizures but also atonic neck seizures (nodding), and stunted growth. OAE is characterised by an onset of seizures between the ages of 3–18 years. Multidisciplinary working groups discussed topics such as how to 1) strengthen the evidence for an association between onchocerciasis and epilepsy, 2) determine the burden of disease caused by OAE, 3) prevent OAE, 4) improve the treatment/care for persons with OAE and affected families, 5) identify the pathophysiological mechanism of OAE, and 6) deal with misconceptions, stigma, discrimination and gender violence associated with OAE.

An OAE Alliance was created to increase awareness about OAE and its public health importance, stimulate research and disseminate research findings, and create partnerships between OAE researchers, communities, advocacy groups, ministries of health, non-governmental organisations, the pharmaceutical industry and funding organizations.

**Conclusions:**

Although the exact pathophysiological mechanism underlying OAE remains unknown, there is increasing evidence that by controlling and eliminating onchocerciasis, OAE will also disappear. Therefore, OAE constitutes an additional argument for strengthening onchocerciasis elimination efforts. Given the high numbers of people with epilepsy in *O. volvulus*-endemic regions, more advocacy is urgently needed to provide anti-epileptic treatment to improve the quality of life of these individuals and their families.

**Electronic supplementary material:**

The online version of this article (10.1186/s40249-018-0400-0) contains supplementary material, which is available to authorized users.

## Multilingual Abstracts

Please see Additional file 1 for translations of the abstract into the five official working languages of the United Nations.

## Background

The filarial nematode *Onchocerca volvulus* is known to cause skin and ocular disease, including blindness, in addition to an excess risk of mortality that increases with microfilarial load [1, 2]. Proposed causes of this excess mortality may be multifarious, including parasite-specific and more generalised immunosuppression [3, 4], in addition to neuro-hormonal involvement, including epilepsy [5]. However, because microfilariae (mf) have never been shown to enter the central nervous system (CNS), onchocerciasis has not been considered to directly cause epilepsy or other brain disorders. However, as early as 1938, Casis Sacre described a syndrome characterized by epileptic seizures, stunted growth and mental retardation in patients with onchocerciasis in Chiapas and Oaxaca States, in Mexico [6]. Since then, a number of studies in Africa have also suggested an association between *O. volvulus* infection and epilepsy [7–10]. In a study in an *O. volvulus*-endemic area in the Mbam Valley in Cameroon, Boussinesq et al. showed that the highest prevalence of epilepsy was observed in villages very close to the Mbam River and in villages with a high community microfilarial load [11]. In the late 1990s, a nodding syndrome epidemic was reported in South Sudan [7] and northern Uganda [12]. Case-control studies revealed an association between nodding syndrome and *O. volvulus* infection [12, 13], but *O. volvulus* infection per se was still not considered to underlie the cause of this syndrome. Moreover, nodding syndrome was thought to occur only in Uganda, South Sudan and Tanzania; nodding syndrome was felt to have a different aetiology than the one driving the epilepsy observed in other onchocerciasis-endemic regions. In 2012, a conference on nodding syndrome was organised by the World Health Organization (WHO) and the Ugandan Ministry of Health in Kampala. During this conference, a case definition for nodding syndrome was proposed, but the problem of the high prevalence of other forms of epilepsy in onchocerciasis-endemic regions was not addressed. In 2015, a follow-up conference organised in Gulu, Uganda, also only focused on nodding syndrome. Recently, several epidemiological studies performed in *O. volvulus*-endemic regions suggested that nodding syndrome should probably be considered as a subset of onchocerciasis-associated epilepsy (OAE) [14] and that OAE may constitute an important but neglected public health problem in many countries where onchocerciasis is still endemic [15]. Therefore, a steering committee was established to organise an international workshop on OAE.

## Main text

### First International Workshop on Onchocerciasis-Associated Epilepsy

From 12–14 October 2017, the first international workshop on onchocerciasis-associated epilepsy (OAE) was held in Antwerp, Belgium (https://www.uantwerpen.be/en/conferences/oae-2017/).

This workshop aimed to: 1) update the knowledge about the different clinical presentations of epilepsy observed in onchocerciasis-endemic regions, 2) create awareness about the public health importance of OAE, and 3) create an OAE Alliance by bringing together organizations and researchers involved in onchocerciasis elimination efforts and those involved in the treatment of and research on epilepsy.

The workshop was attended by 79 participants from 20 different countries, among them 28 African nationals. Participants included a wide variety of experts, among them: neurologists, paediatricians, infectious disease experts, ophthalmologists, epidemiologists, mathematical modellers, parasitologists, onchocerciasis experts, immunologists, pathologists, pharmacologists, anthropologists, public health experts, primary health care specialists and medical entomologists. The workshop was also attended by representatives of the Africa Commission of the International League against Epilepsy, the International Bureau of Epilepsy, and a limited number of representatives of affected communities and of African Ministries of Health. However, despite a broad invitation list (including non-governmental organisations [NGOs] and pharmaceutical companies), only one NGO (Light for the World) attended; none of the pharmaceutical companies producing anti-epileptic treatment were present at the workshop.

The workshop included oral and poster presentations of new research findings and discussions of topics in working groups. The abstract book of the workshop can be found at https://www.uantwerpen.be/en/research-groups/oae/.

### Topics discussed during the workshop

Multi-disciplinary working groups discussed the following topics:

- How to strengthen the body of evidence supporting the association between infection with *O. volvulus* and epilepsy
- What is the burden of disease caused by OAE
- How to prevent OAE
- How to organise surveillance for OAE
- How to improve the treatment/care for persons with OAE and affected families
- How to deal with misconceptions, stigma, discrimination and gender violence associated with OAE
- How to identify the pathophysiological mechanism of OAE
- What should be the components of an OAE prevention and treatment/care policy plan
- How to implement and fund such an OAE policy plan

Main conclusions of the working groups are presented at the end of this paper. Moreover, several papers resulting from the working group discussions will be included in the thematic series on OAE.

### Main research findings presented at the workshop

1. There is a high prevalence and incidence of epilepsy in many *O. volvulus*-endemic regions where onchocerciasis is insufficiently controlled. In recently performed door to door studies in *O. volvulus* meso- and hyperendemic villages close to onchocerciasis vector (blackflies: Diptera, Simuliidae) breeding sites, in the Democratic Republic of the Congo (DRC) [16], Cameroon [17, 18], Tanzania [19], Uganda [20] and South Sudan [21], the prevalence of epilepsy was found to be between 2 and 8%. This rate is much higher than the median epilepsy prevalence in *O. volvulus* non-endemic regions in Africa, which is estimated to be 1.4% [22].
2. In a retrospective cohort study in the Mbam Valley, Cameroon, 5–10-year old children examined in 1991–1993 were grouped into cohorts based on their microfilarial density (0, 1–25, 26–129, > 130 mf/2 skin snips). The cumulative incidence of epilepsy was shown to increase with increasing microfilarial density during childhood [23].
3. For a given microfilarial load, the relative risk of onchocerciasis-associated mortality is statistically significantly higher in younger people (those aged less than 20 years) than in the older age groups (aged 20 years and above) [2]. This cannot be explained by the skin or ocular complications of onchocerciasis but may be explained by OAE-related mortality, given that the peak age of onset of epilepsy in *O. volvulus*-endemic regions is between 3 and 18 years [15] and that children with epilepsy in onchocerciasis-endemic regions experience high mortality rates [24].
4. In *O. volvulus* hyperendemic areas, epilepsy presents within a spectrum of different types of seizures. The most frequent type of seizures is a generalised tonic-clonic seizure. Other forms include: atonic neck seizures (nodding), myoclonic neck seizures, and absence seizures. A minority of individuals have concomitant severe stunting with no delay or delayed external signs of sexual development (the so-called Nakalanga syndrome) [25]. OAE is characterised by an onset of seizures between the age of 3–18 years [15].
5. In several *O. volvulus*-endemic areas with a high prevalence of epilepsy, the annual mass drug administration (MDA) of ivermectin was found not to have been very successful in decreasing epidemiological indicators of *O. volvulus* infection, primarily because of low therapeutic coverages [15, 26]. Modelling studies also confirm that annual MDA may not be sufficient to break *O. volvulus* transmission in areas with very high transmission intensity [26]. Therefore, additional onchocerciasis elimination strategies, including increasing the frequency of treatment (e.g. to 6-monthy) and larviciding of vector breeding sites in fast-flowing rivers, may need to be considered in highly endemic areas.
6. Strengthening ivermectin MDA by increasing coverage and compliance, giving the drug twice a year, and implementation of blackfly control, seem to be the best ways to rapidly decrease the incidence of OAE. In northern Uganda, the incidence of nodding syndrome and other forms of epilepsy started to decrease with the introduction of ivermectin MDA; nodding syndrome cases ceased appearing when a combination of the twice a year distribution of ivermectin together with the deployment of larviciding in the river basins known to contain simuliid breeding sites was implemented [15]. The effect of ivermectin in preventing the onset of OAE is also suggested by recent case-control studies in the DRC showing that persons with epilepsy were less likely to have taken ivermectin compared to age-, sex- and village-matched controls [16, 27], and by an age-shift to older age groups of persons with epilepsy in onchocerciasis-endemic regions where MDA had been implemented for a long time [17, 18].
7. A randomised clinical trial is ongoing in the Logo health zone in the Ituri Province in DRC to evaluate whether ivermectin can decrease the frequency of seizures in persons with OAE [28]. A similar trial in Uganda is investigating whether doxycycline (a tetracycline antibiotic with rigorously quantified macrofilaricidal efficacy via eliminating the worm’s endosymbiotic *Wolbachia* [29]) is able to decrease the frequency of seizures in persons with nodding syndrome [30]. Among patients enrolled in this trial, both asymptomatic and symptomatic *Plasmodium falciparum* malaria infections were associated with poor seizure control [31].
8. The pathophysiological mechanism of OAE is still unknown but might well be a consequence of *O. volvulus-*triggered cross-reactive auto-antibodies. Recently, neurotoxic anti-leiomodin-1 antibodies were detected in patients with nodding syndrome, antibodies that cross-react with *O. volvulus* tropomyosin/troponin [32].
9. Epilepsy-related stigma is wide spread in all *O. volvulus*-endemic regions. Lack of evidence-based awareness campaigns about epilepsy (and its association with *O. volvulus*) means that misconceptions and stigma persevere and are not dealt with. Misconceptions about the origin of the condition influences health-seeking behaviour. Families who have a child with epilepsy, may hide the child at home due to fear of stigmatisation and social exclusion, and turn to traditional healers for treatment, which exacerbates the treatment gap. Women bear a disproportionate burden both as caretakers and patients [33]. Although the research is insufficient, it is well-known to clinicians and social workers that girls with epilepsy are often victims of violence and sexual abuse. The sexual abuse of boys affected by epilepsy is also under-researched. Given the important psycho-social and economic consequences of OAE, the burden of disease caused by OAE appears to be considerable, but the exact magnitude still needs to be determined.
10. There is an urgent need to improve the treatment gap for anti-epileptic treatment and ivermectin coverage to improve patients’ psycho-social and economic well-being. If patients’ seizures are controlled by anti-epileptic drugs, the psychosocial issues and economic prospects of their lives will improve considerably. Workshop attendees, therefore, agreed to establish an OAE policy plan and to test the feasibility and cost effectiveness of this plan in pilot projects.

## Conclusions

Evidence is mounting that infection with *O. volvulus* is associated with a spectrum of epileptic seizures and stunting. However, this body of evidence needs to be strengthened by carefully conducted epidemiological studies.

Given the high prevalence of epilepsy in meso- and hyper-endemic onchocerciasis areas, the burden of disease caused by onchocerciasis needs to be re-estimated taking into account the burden of disease caused by OAE.

Although the exact pathophysiological mechanism of OAE remains unknown, there is increasing epidemiological evidence that by eliminating *O. volvulus* infection, these forms of epilepsy will disappear.

OAE constitutes an additional argument for strengthening onchocerciasis elimination efforts.

Given the high numbers of people with epilepsy in *O. volvulus*-endemic regions, more advocacy is urgently needed to provide anti-epileptic treatment in order to improve the quality of life of those affected and their families.

A community based treatment and care programme for epilepsy needs to be established in remote onchocerciasis endemic regions with a high prevalence of epilepsy. Such a programme should include a surveillance system for epilepsy to allow early diagnosis and treatment and a health education and training programme to decrease misconceptions and stigma related to epilepsy.

Long-term sustainable treatment/care plans for individuals with OAE need to be developed because those affected will continue to suffer even after onchocerciasis has been eliminated.

An OAE Alliance was created to increase awareness about OAE and its public health importance, stimulate research, disseminate research findings and create partnerships between OAE researchers, communities, advocacy groups, ministries of health, NGOs, the pharmaceutical industry and funding organizations.

A follow-up workshop is planned in 2018–19 in Uganda

## Additional file

Additional file 1: Multilingual abstracts in the five official working languages of the United Nations. (PDF 947 kb)

## Abbreviations

CNS: Central nervous system
DRC: Democratic Republic of the Congo
MDA: Mass drug administration
NGO: Non-governmental organisation
OAE: Onchocerciasis-associated epilepsy
WHO: World Health Organization
