# Supplementary material for: Report of the first international workshop on onchocerciasis-associated epilepsy
Source: Infect Dis Poverty. 2018 Mar 22;7:23. doi: 10.1186/s40249-018-0400-0 (PMC5868050; doi:10.1186/s40249-018-0400-0)
Supplement: Supplementary file 1 — Multilingual abstracts in the five official working languages of the United Nations. (PDF 947 kb) [file 40249_2018_400_MOESM1_ESM.pdf]

Translation of the abstract into the six official working languages of the United Nations

تقرير حلقة العمل الدولية الأولى عن الصرع المرتبط بمرض داء كلابية الذنب

روبرت كولبونديرز وميشيل ماندرو وألفريد ك. نجامنشي وميشال بوسينسك و هوتربيكس وجوزيف كامغنو وسارة أونيل وأدريان هوبكنز وباتريك سويكيربويك وماريا-غلوريا باسانيز وروري J. بوست وبيلين بيدريك وبيير ماري بريوكس وويلما A. ستولك، توماس B. نوتمان، ريتشارد إدرو

الملخص
معلومات أساسية:في الآونة الأخيرة، أشارت العديد من الدراسات الوبائية التي أجريت في المناطق التي تتوطنها الذئاب الخبيثة إلى أن الصرع المرتبط بمرض الذنب (داء كلابية الذنب) قد يشكل مشكلة صحية عامة مهمة ولكنها مهملة في العديد من البلدان حيث لا يزال داء كلابية الذنب مرضاً مستوطناً.
النص الرئيسي: في الفترة من 12 إلى 14 أكتوبر 2017، عقدت أول ورشة عمل دولية عن الصرع المرتبطة داء كلابية الذنب (أوي) في أنتويرب، بلجيكا. شهدت الورشة مشاركات قدر عددها ب 79 مشارك من 20 بلداص مختلفاً. وتشير نتائج البحوث الأخيرة بقوة إلى أن هذا المرض أحد الأسباب الرئيسية المسببة للصرع، ولا سيما في المناطق المتوسطة والأوبئة الشديدة لداء كلابية الذنب. إن الإصابة بهذا المرض مع مجموعة من نوبات الصرع، خاصة النوبات الارتجاجية ولكن أيضاً نوبات الرقبة الأتونية (الإيماء)، وتقزم النمو. يتميز الصرع المرتبط بمرض كلابية الذنب بمجموعة نوبات بين أعمار 3-18 سنة. ناقشت الفرق العاملة المتعددة التخصصات مواضيع مثل كيفية 1) تعزيز الأدلة على وجود علاقة بين داء كلابية الذنب والصرع، 2) تحديد عبئ المرض الناجم عن الصرع المرتبط بمرض كلابية الذنب، 3) الحيال دون الإصابة بالصرع المرتبط بمرض كلابية الذنب، 4) تحسين العلاج / الرعاية للأشخاص الذين يعانون من الصرع المرتبط بمرض كلابية الذنب والأسر المتضررة، 5) تحديد الآلية المرضية الفيزيولوجية للصرع المرتبط بمرض كلابية الذنب، و 6) التعامل مع المفاهيم الخاطئة والوصم والتمييز والعنف الجنساني المرتبطة بالصرع المرتبط بمرض كلابية الذنب.
تم إنشاء مجموعة للصرع المرتبط بمرض كلابية الذنب لزيادة الوعي حول هذا المرض وأهمية الصحة العامة، وتحفيز البحوث ونشر نتائج البحوث، وإقامة شراكات بين الباحثين المختصين في الصرع المرتبط بمرض كلابية الذنب والمجتمعات المحلية وجماعات الدعوة ووزارات الصحة والمنظمات غير الحكومية وصناعة المستحضرات الصيدلانية ومنظمات التمويل .
الاستنتاجات: على الرغم من أن الآلية الفيزيولوجية المرضية الكامنة وراء الصرع المرتبط بمرض كلابية الذنب لا تزال غير معروفة، وهناك أدلة متزايدة على أن من خلال السيطرة والقضاء على داء كلابية الذنب، سوف يختفي مرض الصرع المرتبط بمرض كلابية الذنبأيضا. ومن ثم، فإن منظمة الصرع المرتبط بمرض كلابية الذنب تمثل حجة إضافية لتعزيز جهود القضاء على داء كلابية الذنب. نظرا لارتفاع أعداد المصابين بالصرع في المناطق التي-يصيبها المرض هناك حاجة ملحة للمزيد من الدعوة لتوفير العلاج لمكافحة الصرع لتحسين نوعية حياة هؤلاء الأفراد وأسرهم.

Translated from English version into Arabic by Ahmed Alem, through

TRANSLATORS WITHOUT BORDERS

第一届盘尾丝虫病相关性癫痫国际研讨会的报告

Robert Colebunders, Michel Mandro, Alfred K. Njamnshi, Michel Boussinesq, An Hotterbeekx, Joseph Kamgno, Sarah O’Neill, Adrian Hopkins, Patrick Suykerbuyk, Maria-Gloria Basáñez, Rory J. Post, Belén Pedrique, Pierre-Marie Preux, Wilma A. Stolk, Thomas B. Nutman, Richard Idro

摘要

引言：近期在盘尾丝虫病流行区开展的若干流行病学研究表明，在许多仍然流行盘丝虫病的国家，盘丝虫病相关性癫痫(OAE)是一个重要但却被忽视的公共卫生问题。

主要内容：2017 年 10 月 12-14 日，第一届盘尾丝虫病相关性癫痫(OAE)国际研讨会在比利时的安特卫普召开。来自 20 个不同国家的 79 名参会者参加此次研讨会。最新研究结果表明，在盘尾丝虫病中高度流行地区旋盘尾丝虫（*Onchocerca volvulus*）是导致癫痫的重要因素。感染旋盘尾丝虫与癫痫发作的一系列症状相关，主要是全身性强直-阵挛性发作，但同时也包括无力性颈部癫痫发作（点头）和发育不良。OAE 的特征是在 3-18 岁之间癫痫发作。多学科工作组讨论了如下问题：1）加强研究盘尾丝虫病和癫痫之间的关联关系，2）确定由 OAE 引起的疾病负担，3）预防 OAE，4）改善 OAE 患者的治疗/护理以及受影响家庭的生活质量，5）明确 OAE 的病理生理机制，6）处理与 OAE 相关的误解、耻辱、歧视和性别暴力。建立 OAE 联盟旨在提高对 OAE 及其公共卫生重要性的认识，促进研究并传播研究成果，同时在 OAE 研究人员、社区、倡导团体、卫生部、非政府组织、制药业和筹资组织之间建立伙伴关系。

结论：虽然 OAE 的确切病例生理机制仍不清楚，但越来越多的证据表明，通过控制和消除盘尾丝虫病，OAE 也将消除。因此，OAE 是消除盘丝虫病工作的一个重要组成部分。鉴于在旋盘尾丝虫流行地区的癫痫患者人数众多，迫切需要更多的宣传倡导来提供抗癫痫治疗以改善这些患者及其家属的生活质量。

Translated from English version into Chinese by Xue-Jiao Teng, edited by Pin Yang

CHINESE CENTER FOR DISEASE CONTROL AND PREVENTION
NATIONAL INSTITUTE OF PARASITIC DISEASES

Rapport du premier atelier international sur l'épilepsie liée à l'onchocercose (OAE)

Robert Colebunders, Michel Mandro, Alfred K. Njamnshi, Michel Boussinesq, An Hotterbeekx, Joseph Kamgno, Sarah O'Neill, Adrian Hopkins, Patrick Suykerbuyk, Maria-Gloria Basanez, Rory J. Post, Belén Pedrique, Pierre-Marie Preux, Wilma A. Stolk, Thomas B. Nutman, Richard Idro

Résumé

Contexte: Récemment, plusieurs études épidémiologiques menées dans des zones touchées par le ver parasite *Onchocerca volvulus*donnent à penser que l'épilepsie liée à l'onchocercose (OAE) représente un important problème de santé méconnu dans de nombreux pays où l'onchocercose demeure endémique.

Texte principal: Le premier atelier international sur l'épilepsie liée à l'onchocercose (OAE) s'est tenu du 12 au 14 octobre 2017 à Anvers, en Belgique. Cet atelier a accueilli 79 participants venus de 20 pays. De récentes études indiquent clairement que *l'Onchocerca volvulus* joue un rôle majeur dans l'épilepsie, notamment au sein de foyers d'onchocercose mésoendémique et hyperendémique. L'infection provoquée par *l'Onchocerca volvulus* est associée à un ensemble de crises épileptiques, principalement des crises tonico-cloniques généralisées mais aussi des crises atoniques de la région du cou (syndrome du hochement de tête) et un retard de croissance. L'épilepsie liée à l'onchocercose (OAE) se caractérise par une manifestation de crises qui touchent des individus âgés de 3 à 18 ans. Des groupes de travail pluridisciplinaires ont abordé des sujets visant à : 1) conforter les liens de cause à effet entre l'onchocercose et l'épilepsie ; 2) déterminer la charge de morbidité causée par l'OAE ; 3) prévenir l'apparition de l'OAE ; 4) améliorer les traitements/soins dispensés aux personnes souffrant de l'OAE ainsi qu'aux familles affectées ; 5) identifier le mécanisme physiopathologique de l'OAE et 6)

faire face aux malentendus, stigmates, discriminations et problèmes de violence sexiste associés à l'OAE.
L'Alliance OAE a été créée dans le but de mieux faire comprendre l'OAE ainsi que son importance en termes de santé publique, de stimuler la recherche et d'en diffuser les résultats et enfin, de créer des partenariats entre les spécialistes de la recherche sur l'OAE, les communautés, les groupes de défense, les ministères de la santé, les organisations non gouvernementales, l'industrie pharmaceutique et les organisations de financement.
Conclusions: Bien que le mécanisme physiopathologique à la base de l'OAE reste inconnu, il est de plus en plus évident que le combat et l'éradication de l'onchocercose permettrait à terme la disparition de l'OAE. De ce fait, l'OAE constitue un argument supplémentaire pour redoubler les efforts d'éradication de l'onchocercose. Compte tenu du nombre considérable de personnes souffrant d'épilepsie dans les régions atteintes par *l'Onchocerca volvulus*, il faut intensifier les actions de sensibilisation dans le but de fournir des traitements anti-épileptiques qui amélioreront la qualité de vie des individus concernés et de leurs familles.

Translated from English version into French by Elisa Griffin, through

TRANSLATORS
WITHOUT BORDERS

Доклад о первом международном семинаре по эпилепсии, ассоциированной с онхоцеркозом

Роберт Колбандерс, Михел Мандро, Альфред К. Нджамнши, Михел Буссинеск, Ан Хоттербикс, Жозеф Камньо, Сара О'Нил, Эдриан Хопкинс, Патрик Суйкербуйк, Мария-Глория Басаньез, Рори Дж. Пост, Белен Педрик, Пьер-Мари Прё, Вильма А. Столк, Томас Б. Натмэн, Ричард Идро

Аннотация

Краткое описание. Последние эпидемиологические исследования, проведённые в эндемических очагах *Onchocerca volvulus,* дают основание предположить, что эпилепсия, ассоциированная с онхоцеркозом (ЭАО), может представлять собой важную, но обойдённую вниманием общества, проблему здравоохранения в ряде стран, где онхоцеркоз по сей день остаётся эндемическим заболеванием.
Основная часть. С 12 по 14 октября 2017 года в Антверпене, Бельгия прошёл первый международный семинар по эпилепсии, ассоциированной с онхоцеркозом (ЭАО). На семинаре присутствовали 79 участников из 20 разных стран. Результаты последних исследований явно свидетельствуют о том, что *O. volvulus* оказывает существенное влияние на эпилепсию, в особенности в гиперэндемических и мезоэндемических очагах онхоцеркоза. Заражение *O. volvulus* сопровождается спектром эпилептических приступов, в основном генерализованными тонико-клоническими судорогами, а также атоническими судорогами шейных мышц (киванием) и задержкой в росте. Эпилепсия, ассоциированная с онхоцеркозом, характеризуется появлением судорог в возрасте от 3 до 18 лет. Междисциплинарные рабочие группы обсудили следующие вопросы: 1) усиление доказательной базы по взаимосвязи между онхоцеркозом и эпилепсией, 2)

установление бремени заболевания, вызванного ЭАО, 3) профилактика ЭАО, 4) улучшение лечения/медицинского ухода за больными с ЭАО и их семьями, 5) выявление патофизиологических механизмов ЭАО, и 6) устранение заблуждений, отторжения, дискриминации и гендерного насилия, связанного с ЭАО.
С целью повышения осведомлённости о заболевании и его значимости в области общественного здравоохранения, стимулирования научных исследований и распространения их результатов среди учёных, сообществ, групп правозащитников, министерств здравоохранения, НПО, фармацевтической промышленности и финансирующих организаций был создан Альянс по вопросам эпилепсии, ассоциированной с онхоцеркозом (Альянс ЭАО).
Выводы. Несмотря на то, что конкретный патофизиологический механизм, лежащий в основе возникновения ЭАО, остаётся неизвестным, существует всё больше оснований полагать, что при введении контроля за онхоцеркозом и его искоренении также исчезнет эпилепсия, ассоциированная с онхоцеркозом. Следовательно, ЭАО является дополнительным доводом в пользу укрепления усилий по устранению онхоцеркоза. Принимая во внимание значительное число людей, страдающих эпилепсией в эндемических очагах *O. volvulus*, безотлагательно требуется более налаженная система по обеспечению противоэпилептического лечения с целью улучшения качества жизни таких больных и их семей.

Translated from English version into Russian by Liudmila Tomanek and Natalia Potashnik, through

TRANSLATORS
WITHOUT BORDERS

Informe del primer taller internacional de la epilepsia asociada a la oncocercosis

Robert Colebunders, Michel Mandro, Alfred K. Njamnshi, Michel Boussinesq, An Hotterbeekx, Joseph Kamgno, Sarah O'Neill, Adrian Hopkins, Patrick Suykerbuyk, Maria Gloria Basáñez, Rory J. Post, Belén Pedrique, Pierre-Marie Preux, Wilma A. Stolk, Thomas B. Nutman, Richard Idro

Resumen

Antecedentes: Recientemente, varios estudios epidemiológicos realizados en regiones endémicas de *Onchocerca volvulus*han sugerido que la epilepsia asociada a la oncocercosis (OAE por sus siglas en inglés) puede constituir un problema de salud pública importante, aunque se encuentra desantendida en muchos países donde la enfermedad es todavía endémica.
Texto principal: En Octubre 12-14th 2017, se celebró el primer taller internacional sobre epilepsia asociada a la oncocercosis (OAE por sus siglas en inglés) en Amberes, Bélgica. Asistieron al taller 79 participantes de 20 países diferentes. Los resultados de investigaciones recientes sugieren firmemente que *O. volvulus* es un contribuidor importante a la epilepsia, particularmente en áreas meso e hiperendémicas para la oncocercosis. La infección por *O. volvulus* se asocia con un espectro de crisis epilépticas, principalmente crisis tónico-clónicas generalizadas, pero también crisis atónicas del cuello (cabeceo) y retraso en el crecimiento. La OAE se caracteriza por la aparición de crisis epilépticas entre los 3 y los 18 años de edad. Grupos de trabajo multidisciplinarios trataron temas tales como: 1) reforzar la evidencia de una asociación entre oncocercosis y epilepsia, 2) determinar la carga de la enfermedad

causada por la OAE, 3) prevenir la OAE, 4) mejorar el tratamiento/cuidado de las personas con OAE y las familias afectadas, 5) identificar el mecanismo patofisiológico de la OAE, y 6) tratar los conceptos erróneos, el estigma, la discriminación y la violencia de género asociados con la OAE.
Se creó una alianza de la OAE para: aumentar la concientización sobre la OAE y su importancia para la salud pública; estimular la investigación y difundir los resultados de la investigación; y crear asociaciones entre investigadores de la OAE, comunidades, grupos de defensa, ministerios de salud, organizaciones no gubernamentales, la industria farmacéutica y organizaciones de financiación.
Conclusiones: Aunque se desconoce el mecanismo fisiopatológico exacto que subyace a la OAE, cada vez hay más pruebas de que al controlar y eliminar la oncocercosis, la OAE también desaparecerá. Por lo tanto, la OAE constituye una razón adicional para consolidar el trabajo en la eliminación de la oncocercosis. Dado el elevado número de personas con epilepsia en las regiones endémicas con *O. volvulus*, se necesita urgentemente más apoyo para proporcionar tratamiento antiepiléptico que mejore la calidad de vida de estas personas y sus familias.

Translated from English version into Spanish by Ilduara Escobedo and Marcelo G., through

TRANSLATORS
WITHOUT BORDERS
